# The Atypical Cadherin FAT1 Limits Mitochondrial Respiration and Proliferation of Vascular Smooth Muscle Cells

**DOI:** 10.3389/fcvm.2022.905717

**Published:** 2022-05-11

**Authors:** Dario F. Riascos-Bernal, Alishba Maira, Nicholas E. S. Sibinga

**Affiliations:** ^1^Department of Medicine (Cardiology) and Wilf Family Cardiovascular Research Institute, Albert Einstein College of Medicine, Bronx, NY, United States; ^2^Department of Developmental & Molecular Biology, Albert Einstein College of Medicine, Bronx, NY, United States

**Keywords:** FAT1, mitochondria, cell proliferation, vascular injury, restenosis, vascular disease, cell metabolism, oxidative phoshorylation

## Abstract

Smooth muscle cells contribute to cardiovascular disease, the leading cause of death worldwide. The capacity of these cells to undergo phenotypic switching in mature arteries of the systemic circulation underlies their pathogenic role in atherosclerosis and restenosis, among other vascular diseases. Growth factors and cytokines, extracellular matrix components, regulation of gene expression, neuronal influences, and mechanical forces contribute to smooth muscle cell phenotypic switching. Comparatively little is known about cell metabolism in this process. Studies of cancer and endothelial cell biology have highlighted the importance of cellular metabolic processes for phenotypic transitions that accompany tumor growth and angiogenesis. However, the understanding of cell metabolism during smooth muscle cell phenotypic modulation is incipient. Studies of the atypical cadherin FAT1, which is strongly upregulated in smooth muscle cells in response to arterial injury, suggest that it has important and distinctive functions in this context, mediating control of both smooth muscle cell mitochondrial metabolism and cell proliferation. Here we review the progress made in understanding how FAT1 affects the smooth muscle cell phenotype, highlighting the significance of FAT1 as a processed protein and unexpected regulator of mitochondrial respiration. These mechanisms suggest how a transmembrane protein may relay signals from the extracellular milieu to mitochondria to control metabolic activity during smooth muscle cell phenotypic switching.

## Introduction

Vascular smooth muscle cells (SMCs) are the main cellular component of the arterial wall. In healthy mature arteries, SMCs are typically quiescent, non-migratory, and differentiated, as they exhibit high expression of contractile proteins; indeed, their main function is to contract and thus regulate blood vessel diameter or tone. However, in arteries subjected to injury or disease, for instance during atherosclerosis, SMCs dedifferentiate as they lose expression of contractile genes, and they become migratory, proliferative, and synthetic. This phenomenon has been called SMC phenotypic switching (1).

The ability of SMCs to undergo phenotypic switching in the systemic circulation underlies their role in atherosclerosis and restenosis, among other vascular diseases (1, 2). For instance, SMC proliferation and migration contribute to the formation of fibroatheromas; the integrity of the fibrous cap in such lesions relies on SMC survival, proliferation, and synthesis of extracellular matrix (3). These functions are important, as rupture of the fibrous cap may lead to acute myocardial infarction and death (4). Similarly, SMC proliferation contributes to neointima formation that may lead to restenosis after angioplasty and stent placement (5).

Despite the clinical relevance of SMC phenotypic switching, there are no current therapeutic strategies in atherosclerosis or restenosis that specifically and deliberately target this remarkable plasticity, perhaps reflecting our as yet incomplete understanding of this phenomenon. In the last three decades, growth factors and cytokines, extracellular matrix components, regulation of gene expression, neuronal influences, and mechanical forces have been implicated in SMC phenotypic switching (1, 6). Comparatively less is known about cell metabolism in this process.

### SMC Metabolism and Vascular Remodeling

In general, cell proliferation imposes increased requirements for energy and biochemical components, or “building blocks”, but the specific pathways used by different cells to meet these increased demands may differ (7); identification of cell-specific metabolic susceptibilities may guide new therapies. Studies of vascular SMC metabolism in healthy arteries highlight the importance of aerobic glycolysis, glucose oxidation, glycogen turnover, and fatty acid oxidation for the contractile function of SMCs (8, 9). Changes in these metabolic processes have been linked to SMC phenotypic changes. Here we describe a few examples of such studies in different contexts.

Cell culture analyses indicate that platelet-derived growth factor (PDGF), a driver of SMC proliferation, increases glycolytic flux, lactate dehydrogenase (LDH) activity, mitochondrial respiration, and the respiratory reserve capacity of rat aortic SMCs; notably, inhibition of glycolysis limits PDGF-induced SMC proliferation and mitochondrial respiration (10). PDGF also appears to induce mitochondrial fragmentation and fatty acid oxidation (11). Moreover, pharmacological inhibition of LDH reduces glycolysis and PDGF-induced SMC proliferation (12). On the other hand, human coronary artery SMCs proliferating in culture perform both oxidative phosphorylation and aerobic glycolysis to maintain ATP levels, and exhibit high respiratory but low glycolytic reserve capacities (13).

In the setting of vascular injury, SMC proliferation induced by electrical stimulation of rabbit carotid arteries has been associated with increases in both glycolytic and respiratory capacities (14). Balloon injury induces neointimal LDH expression in rat carotid arteries (12) and also upregulates mitochondrial transcription factor A, an essential protein for mitochondrial DNA transcription and replication; notably, knockdown of this factor decreases neointimal growth (15). Similarly, inhibition of hypoxia-inducible factor 1α-which supports the cellular response to hypoxia and induces glycolysis–decreases neointimal formation (16). Likewise, rotenone, an inhibitor of respiratory complex I, decreases neointimal growth after wire injury in mice (17).

In studies of preclinical models and in clinical vascular disease, early work suggests that oxidative phosphorylation is impaired–i.e., uncoupled respiration is prominent–in aortic regions susceptible to atherosclerosis (18) and that a shift in LDH isoform expression compatible with increased glycolysis occurs in human fibroatheromas (19). Similarly, uncoupled respiration in SMCs causes hypertension and increases atherosclerosis in mice (20). In addition, increased mitochondrial DNA damage in mice increases atherosclerosis and plaque vulnerability, and is associated with impaired SMC proliferation; notably, increased mitochondrial DNA damage has been associated with higher-risk plaques and reduced respiratory capacity in humans (21–23). Moreover, preserving mitochondrial DNA integrity increases respiratory reserve capacity, SMC proliferation, and fibrous cap thickness in mouse atherosclerosis (22).

While these studies broadly depict connections between metabolism and vascular remodeling, our understanding of molecular mechanisms that enhance or restrain SMC metabolic activities upon arterial injury or disease is incomplete. In the field of vascular biology, the atypical cadherin FAT1 was identified over 16 years ago through an unbiased strategy to find genes that are up- or down-regulated after arterial injury (24). Subsequent studies of FAT1 in SMC biology have shown that it promotes migration, but limits canonical Wnt signaling, mitochondrial metabolism, SMC proliferation after vascular injury, and neointimal formation (24, 25).

This review focuses on relatively recent advances in understanding of how FAT1 modulates the SMC phenotype, highlighting FAT1 as an unforeseen negative regulator of mitochondrial function. These mechanisms suggest that this transmembrane protein may relay signals from the extracellular milieu to mitochondria to limit respiratory activity during SMC phenotypic switching and to restrict SMC growth.

### FAT1 Is a Transmembrane Protein That Undergoes Proteolytic Processing

FAT1 belongs to the cadherin protein superfamily, which contains over 250 members. In general, cadherins participate in calcium-dependent cell-cell adhesion and are characterized by extracellular cadherin repeat domains (26, 27). The FAT cadherin subfamily comprises enormous single-spanning transmembrane proteins with predicted molecular weights above 500 kDa (27). Specifically, FAT1 structure is remarkable for an extracellular domain bearing 34 predicted cadherin repeats, 5 epidermal growth factor motifs, and a laminin A-G-like motif; the remainder of the protein includes a single hydrophobic transmembrane region and a C-terminal intracellular domain (ICD) with limited homology to that of classical cadherins (24, 27).

**Figure 1:**
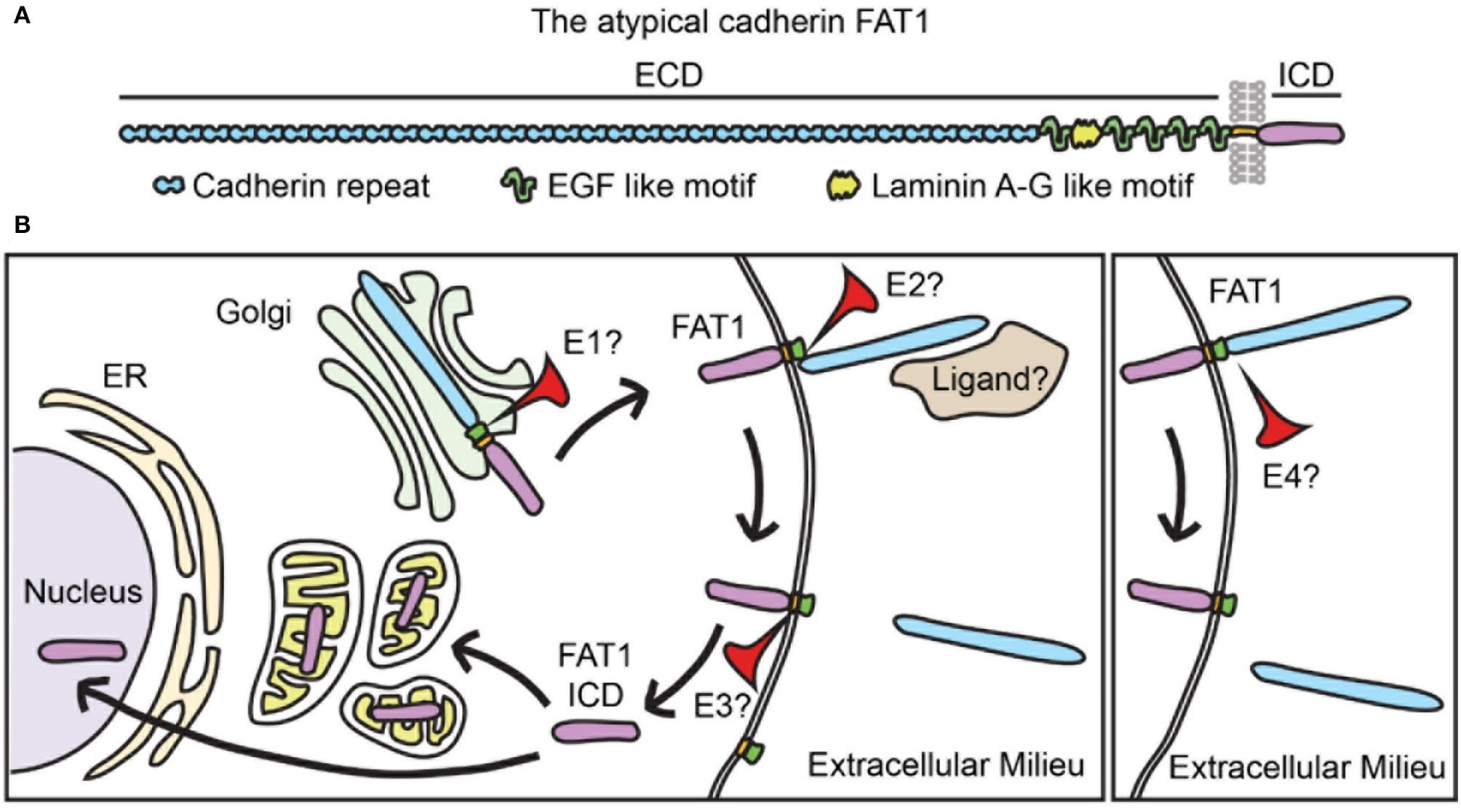
FAT1 is a transmembrane protein that undergoes complex processing. **(A)** FAT1 structure showing its large extracellular domain (ECD) bearing 34 cadherin repeats, 5 EGF-like motifs, and one laminin A-G motif. FAT1 also has a single transmembrane spanning domain and an intracellular domain (ICD). EGF, epidermal growth factor. (**B**) In vascular SMCs the FAT1 ICD has been found in mitochondria and nuclei, suggesting that FAT1 undergoes proteolytic processing that releases the ICD. Studies of FAT1 in cancer biology propose two processing pathways: classical (left panel) and alternative (right panel). The classical pathway involves three sequential proteolytic events: 1) furin-mediated cleavage that occurs in the secretory pathway and results in expression of a heterodimer on the membrane, 2) a disintegrin and metalloproteinase (ADAM)-mediated cleavage of the ECD that results in ectodomain shedding, and 3) a γ-secretase complex-mediated cleavage in the transmembrane region that releases the ICD. In the alternative pathway, intact full-length FAT1 is expressed on the plasma membrane and cleaved in its extracellular domain by an unknown protease. Proteolytic processing has not been formally studied in vascular SMCs, thus whether FAT1 follows classical or alternative proteolytic processing (or both), and the identity of the proteolytic enzymes involved (E1, E2, E3, and E4) remain unknown. Whether FAT1 cleavage is regulated by ligand binding is also an open question (see also refs (30, 31).

Interestingly, the FAT1 ICD has been found in the nucleus and also in mitochondria (see Figure 1) (24, 25, 28). Studies in human cell lines–HEK293 and HeLa cells–support the idea that FAT1 undergoes a regulated proteolytic cleavage that releases its ICD (28). This processing may involve initial ectodomain cleavage by a membrane-associated metalloprotease of the ADAM family, followed by a γ-secretase-mediated event within the transmembrane region that releases the ICD (28), similar to the ligand-dependent processing of Notch. In human pancreatic cancer cells, ADAM10 mediates FAT1 ectodomain shedding (29).

In normal keratinocytes and several cancer cell lines, on the other hand, furin-based FAT1 cleavage forms a heterodimer that is subsequently expressed on the cell membrane, where it is susceptible to ectodomain shedding (30). FAT1 can also undergo a furin-independent proteolytic cleavage that generates a 65 kDa cytoplasmic fragment (31). Interestingly, while keratinocytes show predominant localization of FAT1 to cell-cell junctions, melanoma cells exhibit high levels of cytoplasmic FAT1 expression (31).

Whether similar proteolytic processing of FAT1 occurs in SMCs has not been formally tested; however, the presence of ~60 kDa FAT1 ICD species within mitochondria and frequent cytoplasmic/perinuclear FAT1 distribution in mouse and human SMCs (25) suggests that regulated post-translational proteolysis of FAT1 occurs in these cells. Although overall FAT1 expression increases in response to multiple growth factors strongly implicated in vascular remodeling (24), specific factors or conditions that promote FAT1 processing in SMCs have yet to be identified.

### FAT1 Expression Is High in Settings of Heightened Cell Proliferation

The human *FAT1* gene, initially referred to as *FAT*, was cloned in 1995 from a T-leukemia cell line (32). This pioneering study revealed strong *FAT1* expression in epithelia of the kidney, lung, pancreas, and eye during human fetal development, which are characterized by high rates of cell proliferation. Interestingly, *FAT1* expression was also reported in arterial SMCs in the fetal eye (32).

In contrast, adult human tissues exhibited low levels of *FAT1* in epithelia from the same organs, but higher levels in epithelia of inflamed or neoplastic adult intestinal samples. *FAT1* was also detected in 50% of breast carcinomas studied, with higher mRNA levels corresponding to high-grade cancers (32). *FAT1* expression in adult vascular tissue was not examined or reported in this study.

Studies of rat embryos showed wide expression of *Fat1* during development, notably including the aortic outflow tract (33). The olfactory, oral, intestinal and respiratory epithelium, glomeruli in the kidney, tongue muscles, cartilage, rib and limb buds, and the neuroepithelium of the central nervous system also showed evident *Fat1* expression during development (33). In contrast, *Fat1* was not detected in adult rat tissues except for specific regions of the central nervous system (33).

Mouse developmental studies revealed *Fat1* expression in eight-cell stage embryos before implantation and in a widespread manner after implantation. *Fat1*-positive areas included prospective fore- and mid-brain regions, somites, tail bud, branchial arches, limb buds, facial primordia, lung, dental primordia, ear and eye regions; specific description of the developing vasculature was not provided (34). Notably, in E6.5 embryos, *Fat1* expression was limited to the epiblast, a region of enhanced cell proliferation; at different stages of brain development, *Fat1* expression correlated with areas of high proliferation, and subsided as cells stopped dividing (34).

Subsequent studies are also consistent with the idea that FAT1 is expressed in settings of high cell proliferation. A transformed keratinocyte line, PAM212, shows high FAT1 levels, whereas a relatively differentiated kidney epithelial line, MDCK, exhibits comparatively low levels (35). Immunohistochemistry of breast tumors reveals enhanced FAT1 expression in hyperplasia, metaplasia, and neoplastic transformation; the expression pattern is frequently described as granular cytoplasmic staining (36). Similarly, FAT1 is expressed in oral squamous cell carcinomas and shows a diffuse cytoplasmic and nuclear pattern in poorly differentiated tumors (37). Multiple leukemia cell lines–but not normal peripheral blood or bone marrow cells–express FAT1 (38). Likewise, FAT1 is highly expressed in colon and rectal cancer, mainly on the plasma membrane, and also in adenoma samples, with predominant intracellular pattern, but FAT1 is negligible or not detectable in normal tissues (39). Liver cancer cell lines, but not normal liver tissues, express FAT1 (40).

The identification of splice isoforms indicates additional complexity to patterns of FAT1 expression and possibly function. *Fat1* splice isoforms affecting the ICD are differentially expressed in mouse brain, lung, liver and kidney (41). Notably, subconfluent proliferative rat epithelial cells predominantly express the so-called wild type FAT1, whereas a distinct splice variant predominates in confluent quiescent cells. Nevertheless, total FAT1 expression decreases when cells become quiescent (41). The presence of FAT1 splice isoforms in the vasculature has not been described.

### FAT1 Is Highly Expressed in Injured Arteries That Feature Enhanced SMC Proliferation

Consistent with the aforementioned studies of mammalian development and non-vascular adult tissues and cell lines, the first evaluation of FAT1 expression in the adult vasculature also showed enhanced expression in a setting of heightened cell proliferation (24). Whereas, SMCs are quiescent in normal adult blood vessels, the response of arteries to injury commonly involves, among other features of SMC phenotypic switching, activation and proliferation of SMCs that serve to repair the vessel wall. However, this response may become maladaptive and contribute to excessive intimal growth and subsequent compromise of the arterial lumen, as observed in restenosis and atherosclerosis. The initial assessment of FAT1 expression in the adult rat vasculature found very low signal in normal arteries, but high FAT1 expression in the media and developing neointima of rat carotid arteries after balloon injury (24). Moreover, factors known to promote arterial remodeling, such as angiotensin II, basic fibroblast growth factor, and platelet-derived growth factor, increased FAT1 expression in primary cultured SMCs (24). The effect of angiotensin II was found to involve the type I angiotensin II receptor, activation of nicotinamide adenine dinucleotide phosphate oxidase 1, and subsequent reactive oxygen species production (42). FAT1 expression in SMCs has been observed at the leading edge, cell-cell junctions, nucleus, and perinuclear region (24, 42, 43).

In adult mouse carotid arteries, FAT1 expression is similarly negligible at baseline but high in the media and neointima after arterial injury, wherein FAT1 co-localizes with markers of SMC lineage (25). In particular, FAT1 distribution appears predominantly perinuclear in these ligated arteries, as well as in proliferating mouse and human SMCs in culture. Notably, FAT1 expression is evident in sections of restenotic human coronary arteries adjacent to sites of previous stent placement, and coincides with SMC marker expression in many areas (25). Interestingly, the apparent perinuclear pattern of FAT1 expression in proliferating mouse and human SMCs likely corresponds at least in part to the presence of FAT1 fragments in mitochondria, as indicated by confocal imaging and cell fractionation studies, discussed further below.

### FAT1 Works as a Brake on SMC Proliferation Upon Vascular Injury

Although FAT1 expression in SMCs is increased in remodeling arteries characterized by high levels of SMC proliferation and induced by growth factors known to promote SMC mitogenesis (24, 25), both *in vitro* and *in vivo* loss-of-function as well as *in vitro* gain-of-function studies support the idea that FAT1 opposes SMC proliferation and therefore restricts one of the hallmarks of SMC phenotypic switching. In primary mouse aortic SMCs, siRNA-mediated knockdown of FAT1 increased both cyclin D1 expression, a marker of cell cycle activation, and DNA synthesis, indicating that FAT1 limits cell cycle progression in SMCs. On the other hand, expression of the FAT1 ICD in SMCs was sufficient to limit DNA synthesis and cell growth in culture (24). Consistent with these observations, genetic inactivation of *Fat1* in SMCs using the mouse Cre-LoxP system increased cell proliferation in culture, while restoration of FAT1 ICD expression in SMCs otherwise lacking FAT1 was sufficient to reduce cyclin D1 levels (25). Moreover, siRNA-mediated knockdown of FAT1 in primary human aortic SMCs also increased cyclin D1 protein levels and DNA synthesis (25).

Global inactivation of the *Fat1* gene in mice leads to perinatal lethality, attributable to glomerular alterations, renal dysfunction, and cranial midline defects (44). No developmental vascular phenotype was reported in this study; similarly, characterization of adult mice with conditional loss of SMC FAT1 showed normal-appearing vascular structure. Interestingly, these mice lacking SMC FAT1 showed an accelerated and more robust response to arterial injury, characterized by markedly increased medial SMC hyperplasia and neointimal growth associated with enhanced expression of phospho-histone H3 and cyclin D1, markers of cell proliferation (25). Consistent with findings in cell culture, this result suggests that FAT1 acts as a physiological brake on SMC growth during the response to arterial injury.

FAT1-mediated control of cell proliferation is not limited to SMC biology. Global genetic inactivation of *Fat1* in mice results in developmental cranial defects in neural tube closure associated with enhanced proliferation of cortical precursors; moreover, *in utero* knockdown of *Fat1* in cortical precursors results in higher proliferation of radial glial precursors (45). Likewise, loss of FAT1 in mice leads to fully penetrant lens epithelial defects during development characterized by increased epithelial cell proliferation (46). In addition, frequent inactivation of FAT1 due to mutations or deletions in humans is associated with esophageal squamous cell carcinoma, oral squamous cell carcinoma, astrocytoma, glioblastoma, colorectal cancer, and head and neck cancer (47–52).

### FAT1 Inhibits β-Catenin-Mediated Transcriptional Activity in SMCs

**Figure 2:**
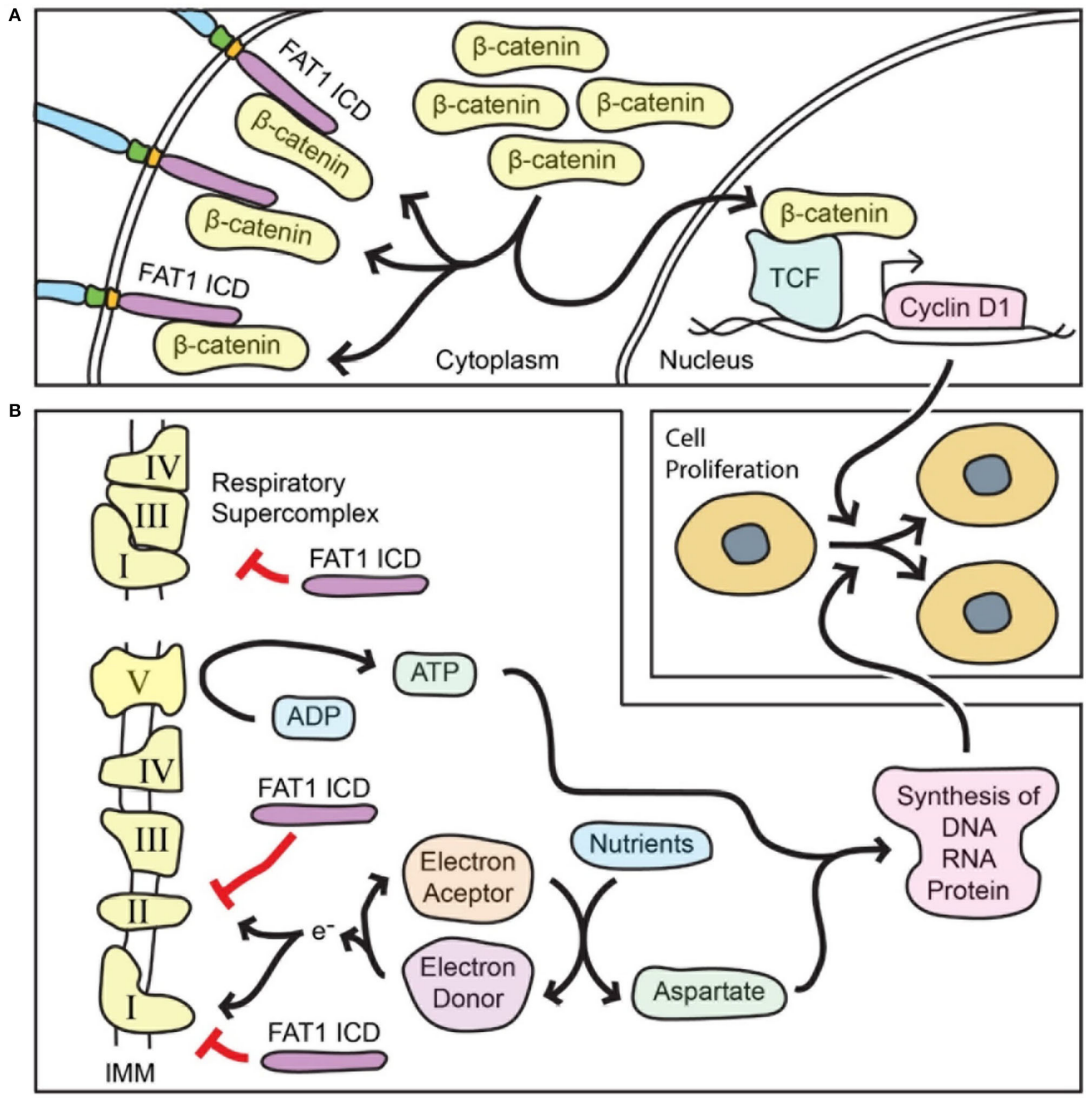
FAT1 limits both β-catenin-mediated transcriptional activity and mitochondrial respiration in order to restrict vascular SMC proliferation. (**A)** The FAT1 ICD interacts with β-catenin and decreases β-catenin accumulation in the nucleus. In general, nuclear β-catenin interacts with the TCF family of transcription factors to active transcription of target genes that promote cell proliferation such as the gene for cyclin D1, a key promoter of cell cycle progression. Thus, FAT1 suppresses the pro-proliferative function of β-catenin. (**B)** The FAT1 ICD within mitochondria decreases the activity of respiratory complexes I and II, and the formation of respiratory supercomplexes; both of these effects reduce mitochondrial respiration. The respiratory function is essential for replenishing electron acceptors that in turn are necessary for aspartate biosynthesis from nutrients. Respiration also results in ATP synthesis. Both ATP and aspartate are essential for synthesis of macromolecules that are required for cell division. Thus, FAT1 suppresses the pro-proliferative function of mitochondrial respiration. IMM, inner mitochondrial membrane.

The molecular mechanisms underlying FAT1-mediated growth regulation are thus of interest for vascular biology and for other fields. As the non-redundant central effector of canonical Wnt signaling, β-catenin has been broadly associated with cell proliferation in both development and neoplasia. In SMCs, β-catenin promotes proliferation and survival and is essential both for arterial wall formation during development and for neointimal growth after arterial injury in adulthood (53, 54). The FAT1 ICD shares limited homology with β-catenin-interacting sequences found in classical cadherins (24, 32), and endogenous FAT1 and β-catenin can interact in SMCs, likely via two domains within the FAT1 ICD (24). Notably, the level and form of FAT1 expression affected localization and function of β-catenin within cells: *Fat1* knockdown increased β-catenin nuclear accumulation and transcriptional activity, while overexpression of a FAT1 ICD directed to the cytoplasm, but not to the nucleus, blocked these effects (summarized in Figure 2A). Thus, in addition to partial conservation of structure, FAT1 shares some functional similarities with classical cadherins, though it appears less efficient than E-cadherin as an inhibitor of β-catenin activity (24). Loss of FAT1-mediated control of β-catenin signaling (24) was subsequently shown to occur in some cancers with FAT1 mutations (50).

### FAT1 Restrains Mitochondrial Respiration in SMCs

Arterial injury in mice lacking FAT1 in SMCs leads to a marked increase in proliferation of these cells, raising the possibility that FAT1 works via additional anti-proliferative mechanisms beyond its effects on β-catenin. Pursuit of this idea led to an unbiased proteomic approach to look for novel FAT1 ICD interactors, which in turn revealed an unforeseen FAT1 function in mitochondria (25). Surprisingly, tandem affinity purification followed by mass spectrometry identified mitochondrial proteins, including several associated with the inner mitochondrial membrane, as candidate FAT1 ICD interactors. Co-immunoprecipitation studies validated FAT1 interaction with NDUFS3, a respiratory complex I subunit, and with prohibitin, a regulator of complex I. Importantly, fractionation of primary aortic SMCs revealed FAT1 fragments in mitochondria, including an ~60 kDa FAT1 protein specific to mitochondria, while confocal imaging demonstrates co-localization of FAT1 and mitochondria following a perinuclear distribution (25).

Evaluation of mitochondrial respiration showed that loss of FAT1 increased basal, ATP-linked, and maximal oxygen consumption in mouse and human primary SMCs; notably, expression of a form of FAT1 ICD exclusively targeted to mitochondria was sufficient to return these parameters toward control levels (25). Loss of FAT1 also increased levels of aspartate; this amino acid is essential for macromolecular synthesis and cell proliferation, and its biosynthesis requires electron acceptors produced during mitochondrial respiration (55, 56). Notably, inhibition of respiration with rotenone, a chemical inhibitor of respiratory complex I, suppressed the growth advantage of SMCs lacking FAT1, and knockdown of NDUFS3, a complex I subunit, reduced DNA synthesis of FAT1-deficient SMCs toward control levels (25).

Further mechanistic studies showed that the FAT1 ICD interacts with immunocaptured respiratory complexes I, II and V, and that loss of FAT1 increases the enzymatic activity of respiratory complexes I and II. Moreover, SMCs lacking FAT1 exhibited enhanced formation of respiratory supercomplexes (25), which are thought to facilitate electron transport without increasing production of reactive oxygen species (summarized in Figure 2B) (57, 58).

Altogether, these studies suggest that FAT1 ICD fragments localize to mitochondria and restrain respiration by limiting mitochondrial complex I and II activities, and by impeding complex I incorporation into respiratory supercomplexes. In turn, this FAT1-mediated restraint on mitochondrial respiration restricts SMC proliferation, at least in part by limiting the availability of aspartate, a metabolite essential for cell division (25).

## Concluding Remarks

As a transmembrane protein, FAT1 is positioned to serve as a relay for signals from the extracellular environment to the cell interior. The studies cited above support the idea that increased SMC FAT1 expression after arterial injury, due in part to growth factors including but not limited to angiotensin II, serves as a physiological brake on SMC proliferation by limiting both β-catenin transcriptional activity and mitochondrial respiration (24, 25, 42). The net result of these functions is to prevent excessive neointimal growth and vascular occlusion, and arguably, to promote efficiency of vascular repair (25).

These processes may incorporate critical points that are susceptible to therapeutic intervention in cardiovascular disease, yet our understanding of FAT1 in vascular biology is far from complete. Pertinent gaps of knowledge include: a) the function of the enormous FAT1 extracellular domain, including the identity of any ligand(s), b) processing mechanisms that lead to FAT1 ICD release and transport to mitochondria, c) *in vivo* sufficiency of mitochondrial FAT1 ICD species to limit cell proliferation and vascular remodeling, d) effects of FAT1 on cell metabolism beyond ATP and aspartate availability, e) the importance of FAT1 for SMC migration–previously characterized *in vitro* (24, 42, 43)–in the *in vivo* setting, and f) the significance of FAT1 in vascular diseases such as atherosclerosis, restenosis, or aneurysm formation.

Mechanistic insights into the FAT1-mediated regulation of SMC activities, including mitochondrial function and cell metabolism, may extend beyond cardiovascular biology to impact other areas of great importance for human health, notably renal disease and cancer biology. Inherited FAT1 mutations affect kidney development and have been linked to clinical glomerulotubular nephropathy (59), while somatic mutations are increasingly recognized in both solid tissue and hematopoietic cancers (27). FAT1 cellular expression patterns in some instances include a suggestive cytoplasmic or perinuclear distribution (31, 36, 37, 39), but whether FAT1 localizes to and/or functions in mitochondria in cell types other than SMCs has not been reported. In view of emerging interest in mitochondrial contributions to macromolecular synthesis and oncometabolite production that support cancer cell growth and phenotype (60), such mechanisms could be of considerable interest for FAT1-related pathology both within and outside of vascular biology.

## Author Contributions

DFR-B, AM, and NESS reviewed the literature, conceived, and wrote the manuscript. All authors contributed to the article and approved the submitted version.

## Funding

DR-B is supported by an American Heart Association Career Development Award, 19CDA34660217. AM is supported by NIH T32HL144456. NS is funded by NIH awards R01HL149921 and R21NS16480 and American Heart Association awards 19TPA34890070 and 20TPA35490392.

## Conflict of Interest

The authors declare that the research was conducted in the absence of any commercial or financial relationships that could be construed as a potential conflict of interest.

## Publisher's Note

All claims expressed in this article are solely those of the authors and do not necessarily represent those of their affiliated organizations, or those of the publisher, the editors and the reviewers. Any product that may be evaluated in this article, or claim that may be made by its manufacturer, is not guaranteed or endorsed by the publisher.
